# Case Report: Upper extremity deep vein thrombosis revealing an occult invasive ductal breast carcinoma

**DOI:** 10.3389/fcvm.2026.1742549

**Published:** 2026-02-03

**Authors:** Stefan Chiorescu, Ruxandra Oiegar, Mihaela Mocan, Mihaela Trif, Razvan Alexandru Ciocan, Roxana Mihaela Chiorescu

**Affiliations:** 1 Department of Surgery, Iuliu Hațieganu University of Medicine and Pharmacy, Cluj-Napoca, Romania; 2 Department of Surgery, Emergency Clinical County Hospital, Cluj-Napoca, Romania; 3 Department of Internal Medicine, Department of Cardiology Rehabilitation, Iuliu Hațieganu University of Medicine and Pharmacy, Cluj-Napoca, Romania; 4 Doctoral School, Iuliu Hațieganu University of Medicine and Pharmacy, Cluj-Napoca, Romania; 5 Internal Medicine Department, Iuliu Hatieganu University of Medicine and Pharmacy, Cluj-Napoca, Romania; 6 Department of Internal Medicine, Emergency Clinical County Hospital, Cluj-Napoca, Romania; 7 Medical Oncology Department, Emergency Clinical County Hospital, Cluj-Napoca, Romania; 8 Department of Surgery — Practical Abilities, Iuliu Hatieganu University of Medicine and Pharmacy, Cluj-Napoca, Romania

**Keywords:** anticoagulant treatment, breast carcinoma, cancer-associatedvenous thromboembolism, deep vein thrombosis, upper limb

## Abstract

Deep vein thrombosis of the upper limb is a rare but clinically significant condition, as it may represent the first manifestation of an underlying malignancy and often poses diagnostic and therapeutic challenges. We present the case of a 66-year-old woman with no significant medical history, admitted for extensive deep vein thrombosis of the left upper limb that developed shortly after surgical decompression of the carpal tunnel. Postoperatively, she presented with marked swelling of the left arm, and Doppler ultrasonography confirmed thrombosis of the deep venous system. Thoracic computed tomography illustrated tumoral infiltration of the left subclavian vein and artery, causing segmental venous thrombosis and severe arterial stenosis. Despite elevated serum levels of the tumor marker Cancer Antigen 15-3, both mammography and breast ultrasonography were negative. A thoracic computed tomography–guided biopsy established the diagnosis of invasive lobular breast carcinoma, Nottingham grade 1, estrogen receptor–positive, with Ki-67 < 5%. Staging procedures included positron emission tomography combined with computed tomography, magnetic resonance imaging of the breast, and confirmatory breast biopsy. The particularity of this case lies in its atypical onset, initially mimicking carpal tunnel syndrome, combined with upper limb venous thrombosis caused by an occult breast tumor undetectable by conventional imaging but infiltrating the axillary vascular bundle. Therapeutic decisions followed international guidelines for cancer-associated thrombosis, with anticoagulation tailored to bleeding risk and oncologic therapy individualized according to the tumor's immunologic profile.

**Conclusions:**

Upper extremity deep vein thrombosis is frequently associated with malignancy and may represent its first clinical manifestation, as in our patient, whose axillary breast carcinoma invaded the arteriovenous structures without detectable breast lesions. Early recognition of this condition is essential for timely diagnosis and improved prognosis. Direct oral anticoagulants represent a valuable therapeutic option with multiple advantages. Their potential antineoplastic effects—particularly those of factor XI inhibitors currently under investigation—may provide additional benefits in cancer-associated thrombosis.

## 1 Introduction

Deep vein thrombosis of the upper extremity is an uncommon in comparison to deep vein thrombosis of the lower extremity but clinically significant condition, most frequently associated with central venous catheters, trauma, or malignancy.

The incidence of upper extremity deep vein thrombosis (UEDVT) has increased in recent years, accounting for 4%–10% of all venous thromboembolic events. The main risk factors include the presence of central venous catheters, peripherally inserted central catheters, infections, and malignancy—particularly breast cancer (1–3). Although pulmonary embolism occurs less frequently than in lower limb thrombosis (2%–5% vs. 16%–28%), UEDVT remains clinically relevant, due to complications such as limb pain, edema, post-thrombotic syndrome, and limitations in intravenous therapy (3–5).

The highest incidence occurs in individuals aged 60–69 years, with hematologic malignancies, gastrointestinal (GI) and pancreatic tumors, lung and breast cancer being common predisposing conditions. In cancer patients, the use of central venous catheters for chemotherapy further increases the risk (3–6).

Systemic inflammation also contributes to thrombus formation through cytokines such as tumor necrosis factor-α and interleukins 6 and 8, which activate leukocytes and platelets, promote endothelial dysfunction, and inhibit natural anticoagulant pathways (1, 7, 8). Thrombophilia is another predisposing factor for UEDVT; the most frequent entities include factor V Leiden mutation, prothrombin gene mutation (G20210A), Anti-thrombin III defficiency, protein C and protein S deficiencies, and anti-phospholipid antibodies (9).

Another etiology is venous thoracic outlet syndrome, in which mechanical compression of the subclavian vein leads to repetitive endothelial injury and thrombosis of the axillary–subclavian segment (9).

Despite progress in managing venous thromboembolism, evidence regarding UEDVT—particularly in cancer patients—remains limited. Most trials and guidelines address lower limb thrombosis, leaving therapeutic strategies for upper extremity events uncertain. Direct oral anticoagulants (DOACs) are increasingly preferred over low-molecular-weight heparins (LMWHs) for cancer-associated thrombosis (CAT). Still, their efficacy and safety in upper-limb thrombosis, especially in solid tumors such as breast cancer, remain unclear. New potential targeted agents (factor XI inhibitors) are currently being investigated for anticoagulation therapy, especially in oncologic patients.

This case highlights the importance of early recognition of UEDVT, given its association with potentially severe entities that may otherwise go undetected by conventional investigation tools. It also underlines the diagnostic and therapeutic challenges for anticoagulation in CAT.

## 2 Case report

We report the case of a 66-year-old female, without relevant past medical history, in whom the current disease had insidiously started three months before presentation with paresthesia of the left forearm and the last two fingers, symptoms that were initially interpreted as carpal tunnel syndrome.

The patient presented to the Emergency Department (ED) for increasing volume of the left upper extremity, associated with intense pain, which appeared suddenly three days after the surgical intervention for carpal tunnel syndrome decompression.

On clinical examination, the patient presented with grade I obesity, with normal-colored skin and mucosa, preserved oxygen saturation (98% in ambient air), rhythmic heart sounds without murmurs, blood pressure of 141/93 mmHg, and heart rate of 100 bpm. The left upper extremity was markedly edematous. No palpable mass is detected in the left axillary cavity.

The electrocardiogram showed sinus rhythm, HR 100 bpm, intermediate QRS axis, without significant ST-T alterations or conduction abnormalities. Laboratory findings revealed leukocytosis with neutrophilia, mild normocytic normochromic anemia, elevated C-reactive protein (CRP) and D-dimers (596 ng/mL, reference values for D-dimers < 500 ng/mL), while renal and hepatic functions were within normal limits.

A venous Doppler examination performed in the ED demonstrated complete thrombosis of the left basilic and brachial veins, extending into the subclavian vein. She was admitted to the Internal Medicine Department with the diagnosis of left UEDVT. Consequently, anticoagulant therapy with LMWH (enoxaparin 1 mg/kg bid, subcutaneously) was initiated.

**Figure 1:**
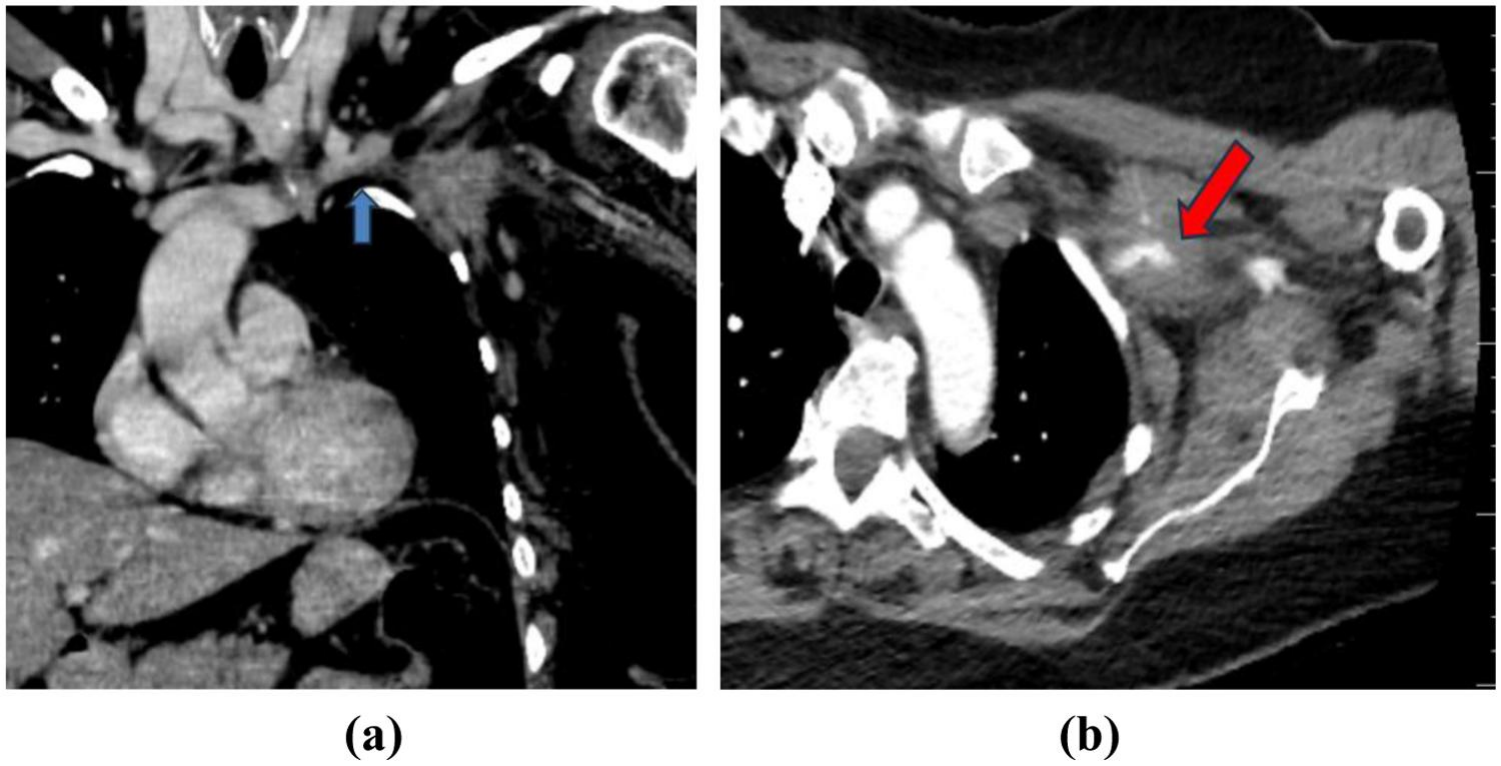
**(a)** Chest CT coronal section with IV contrast—venous time—arrow indicates thrombus in the left subclavian vein, in continuity with the tumor (invasion/tumor thrombus). **(b)** Chest CT axial section with IV contrast—arterial time –arrow indicates the embedded subclavian/axillary artery, still permeable.

A reevaluation with Doppler ultrasound confirmed extensive thrombosis of the subclavian, axillary, and brachial veins, as well as an irregular, heterogeneous axillary mass compressing these vessels. Given the clinical and paraclinical data, a thoracic Computed Tomography (CT) Angiography was performed that identified a 38 × 52 × 58 mm iodophile mass localized at the apex of the left axilla, infiltrating the pectoralis minor and intercostal muscles. Moreover, the images illustrated tumoral infiltration of the left subclavian vein and artery causing segmental vein thrombosis and respectively, severe arterial stenosis. Multiple suspicious adenopathies were present, along with a spiculiform pulmonary nodule in the right upper lobe and hypovascular hepatic lesions (likely hemangiomas) (Figure 1). In this context, tumoral markers were dosed in order to determine the type of tumor.

**Figure 2:**
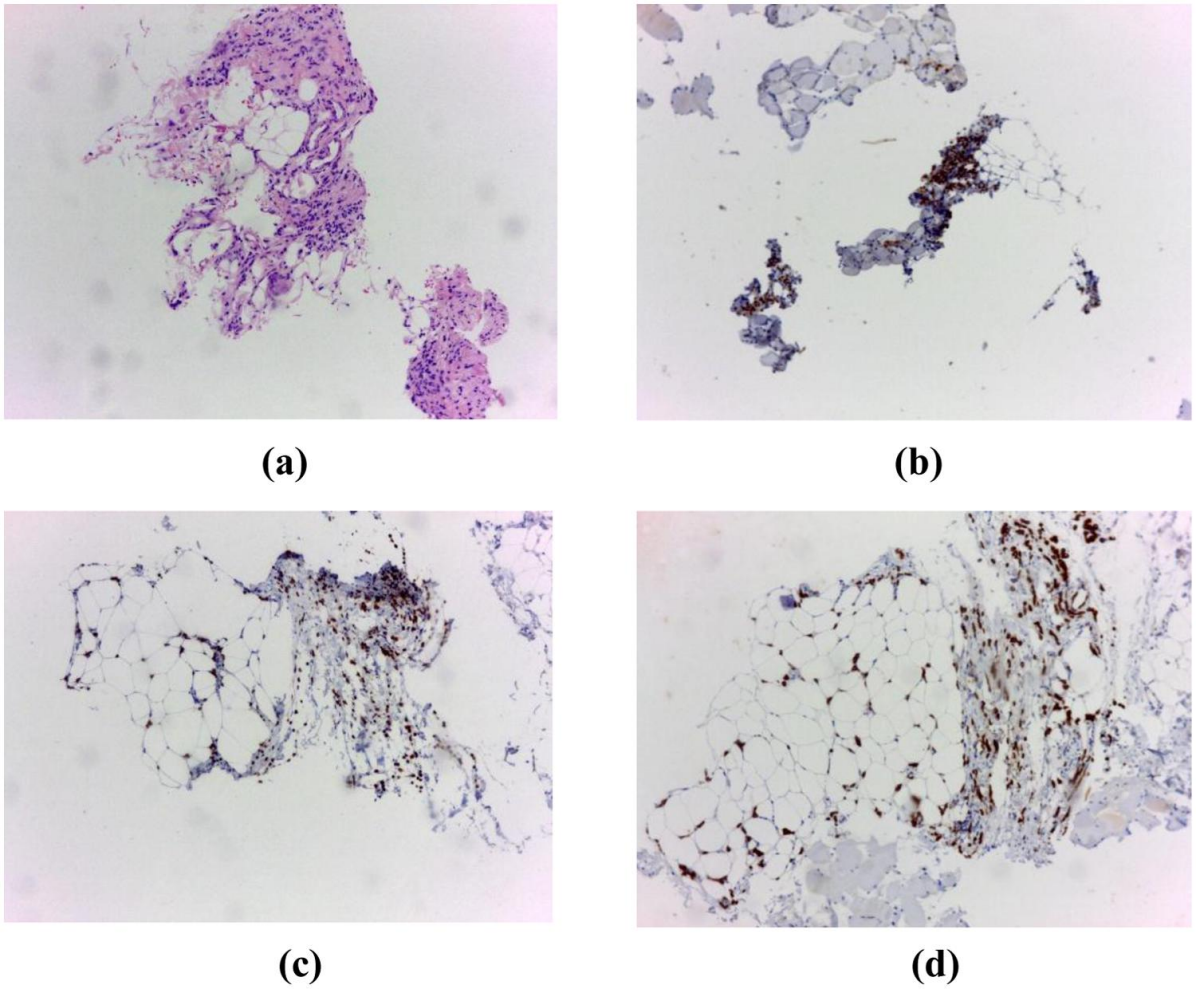
**(a)** Hematoxylin and eosin (H&E) staining, 10× objective. Breast tissue fragments infiltrated by an invasive lobular carcinoma, **(b)** immunohistochemistry for Estrogen Receptor (ER), **(c)** immunohistochemistry for GATA3, **(d)** immunohistochemistry for pancytokeratin (pCK).

Tumoral marker Cancer Antigen (CA) 15-3 was significantly elevated at 991.6 U/mL (reference values for CA 15-3 < 31,3 U/mL). Despite these findings, mammography and breast ultrasonography were negative, with a Breast Imaging Reporting and Data System (BI-RADS) score of 2. A CT-guided biopsy of the axillary mass revealed an invasive lobular breast carcinoma, Nottingham grade 1, 100% Estrogen Receptor—positive, Progesterone Receptor—negative, with a marker of proliferation Kiel 67 (Ki-67) index < 5% (Figure 2).

For complete staging, the case was presented to the Tumor Board, which recommended breast Magnetic Resonance Imaging (MRI) to identify the primary tumor, Positron Emission Tomography/Computed Tomography (PET-CT) to evaluate possible metastasis, and an additional biopsy to determine Human epidermal growth factor receptor (HER2) status.

The breast MRI showed a left axillary tumor with malignant aspects, measuring 48/47 mm, with a spiculiform border, invading the deep surface of the small pectoralis muscle, with extension to the tip of the axilla. In addition, there was a 7 mm nodular lesion in the superior external quadrant of the left breast, without contrast enhancement. Multiple suspicious bilateral axillary adenopathies were observed. The final MRI classification of the mass was BI-RADS 5 (highly suggestive of malignancy).

**Figure 3:**
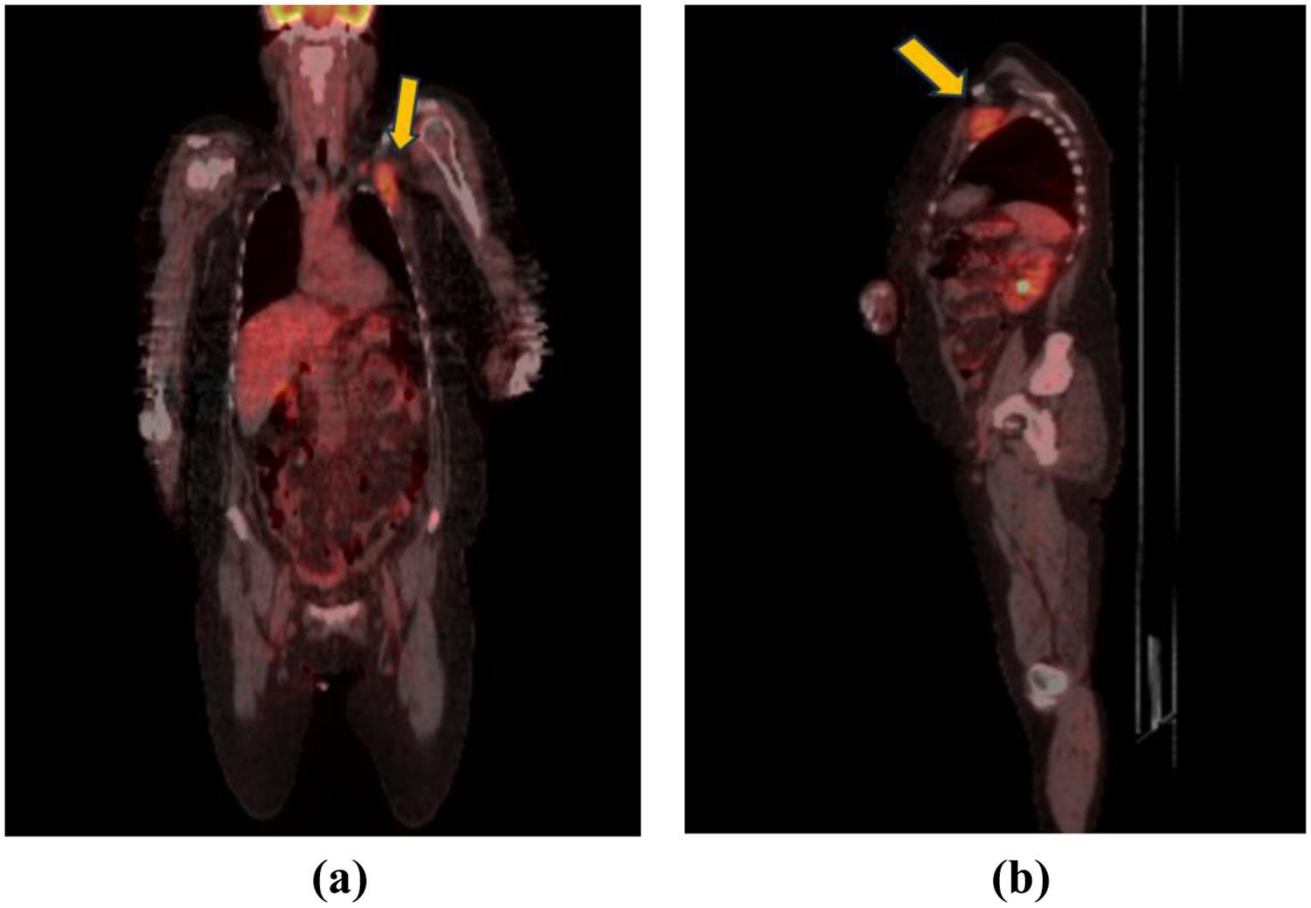
**(a,b)** PET—CT image—hypersensitive interpectoral formation.

The PET-CT examination showed a metabolically active left subclavian mass infiltrating the muscle and the left clavicular artery and vein, with associated supraclavicular lymphadenopathies (Figure 3). In addition, multiple osteolytic bone lesions were detected, some with high fludeoxyglucose activity and highly suspicious, which will also require close follow-up in the future.

The patient received anticoagulation treatment for the deep vein thrombosis. While admitted, she was treated with LMWH (enoxaparin 1 mg/kg bid, sc) for 7 days; afterwards, on discharge, she received treatment with DOAC—Rivaroxaban 15 mg bid, for 14 days and then 20 mg od. Oncological treatment consisted of hormone therapy with an aromatase inhibitor (letrozole). Following Tumor Board recommendations, a supplementary guided biopsy was performed, which indicated a HER2-positive cancer. The Oncology Team Board further recommended fluorescence *in situ* hybridization (FISH) for targeted treatment.

On follow-up, two months after the UEDVT, with continuous anticoagulation therapy (Rivaroxaban), the patient is no longer symptomatic, and the limb edema has resolved completely. On ultrasound, the thrombus has significantly shrunk, with a permeable subclavian vein. Regarding the severe arterial stenosis, an interventional approach (balloon dilation +/- stent) or a surgical one (arterial by-pass) was considered. However, given the asymptomatic patient with a present peripheral pulse, we decided on conservative treatment and close monitoring. The patient remained on anticoagulation therapy with DOAC for an unlimited time.

**Figure 4:**
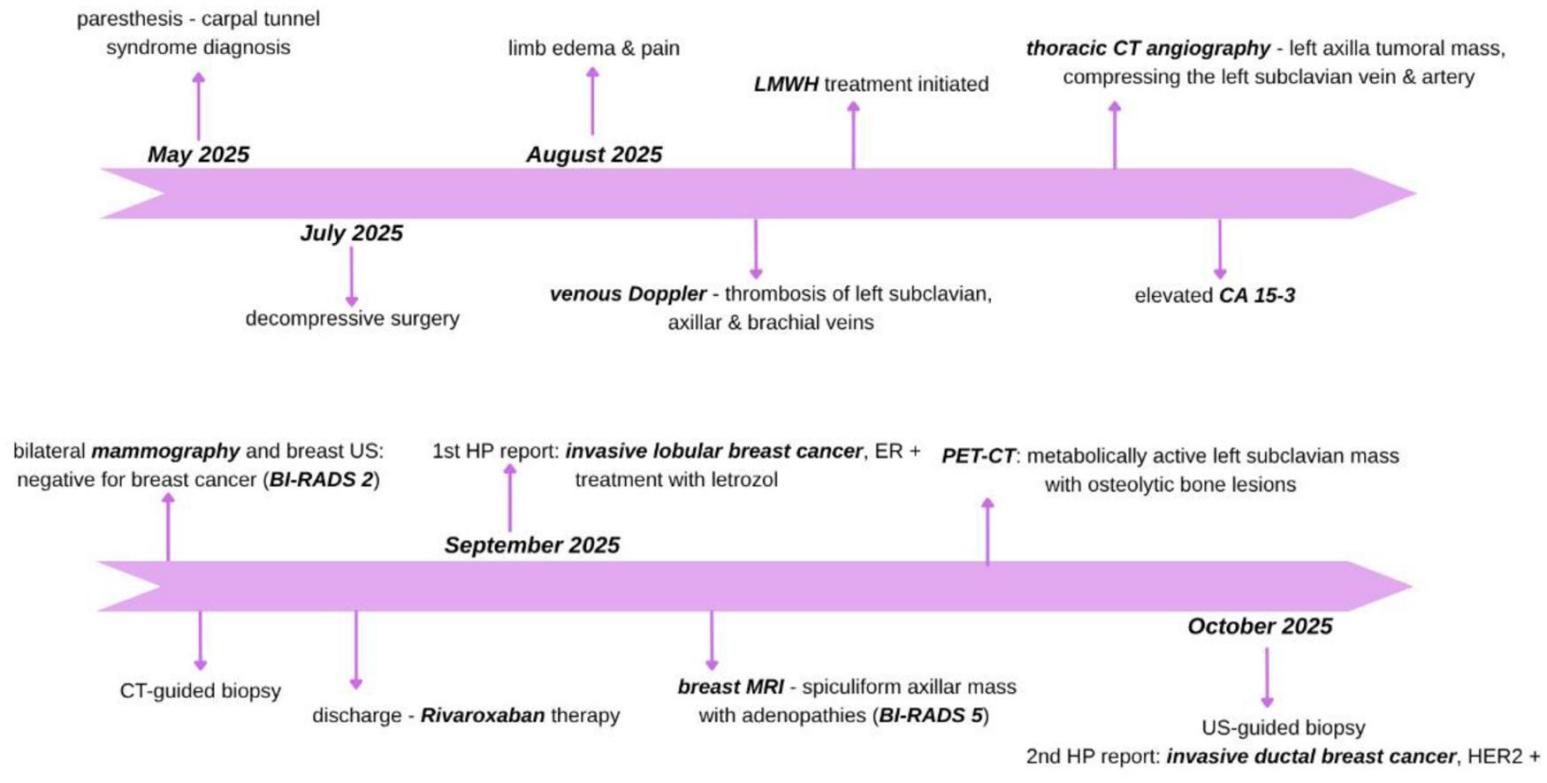
Case timeline.

Regarding the oncologic pathology, the patient is closely evaluated by specialists, undergoing hormonal therapy with letrozole. The case timeline is presented in Figure 4.

As for prognosis, there is an advanced breast carcinoma; however, due to only bone metastasis, the patient is included in the “most favorable” metastatic group. In addition, the tumor has a slowly progressive histology, with a Ki-67 proliferation index < 5% and a negative HER2 status (no HER2 gene amplification on FISH). In patients with similar tumors, following treatment with letrozol (an aromatase inhibitor) and palbociclib (a CDK4/CDK6 inhibitor), the progression-free survival rate is around 27 months, while the overall survival rate is around 51 months. Therefore, the patient has a relatively good general prognosis despite the advanced pathology. Regarding functional prognosis, the patient has progressively regained mobility of the left upper extremity.

Even though the tumor included the left subclavian artery, the patient did not encounter any bleeding complications while on anticoagulation therapy.

## 3 Discussion

### 3.1 Particularities of the case

This case presents several particularities, which highlight the diagnostic and clinical complexity of the condition. The initial manifestation was atypical, consisting of paresthesia in the left upper limb, most likely secondary to brachial plexus compression, leading to an initial interpretation of carpal tunnel syndrome.

Another unusual aspect of this case was the thrombotic presentation. The uncommon localization at the upper limb was associated with mechanical compression rather than systemic hypercoagulability. In this context, the likely mechanism involved extrinsic venous compression and local stasis produced by the underlying tumor mass. Bearing in mind this particularity is of paramount importance, as UEDVT could represent a paraneoplastic manifestation or the first clue to an occult malignancy.

A further point of interest lies in the discordant diagnostic findings. Despite negative mammography and breast ultrasonography, the serum CA 15-3 biomarker was markedly elevated. This discrepancy illustrates one of the significant challenges in clinical oncology—relying solely on imaging modalities may not always detect specific tumor localizations, particularly in the presence of infiltrative or non-mass-forming lesions. Ultimately, in this case, histopathological examination provided the definitive confirmation of malignancy, emphasizing its essential diagnostic role over imaging techniques.

Taken together, these findings underscore the importance of a multidisciplinary approach that integrates clinical, imaging, and laboratory data to avoid diagnostic delays in atypical cancer presentations.

Another UEDVT case, associated with malignancy, was treated similarly with a factor Xa inhibitor (apixaban). The patient was a 43-year-old male without previous medical history, presenting with spontaneous mild swelling of the left upper limb. Given the young age, screening for thrombophilia was performed, identifying a heterozygous Factor V Leiden mutation (G1691A), associated with a 5- to 7-fold risk of thrombosis. To evaluate for possible venous compression, the team performed a thoracic contrast-enhanced CT, which identified a mediastinal mass, later confirmed as a malignant mediastinal teratoma. The oncologic treatment consisted of chemotherapy and surgical removal, while anticoagulation treatment with DOAC (apixaban) was continued until surgery and interrupted six weeks after the end of chemotherapy. There were no other VTE events during the first year of follow-up or while the patient was on systemic therapy (10).

UEDVT can be caused by malignant masses outside the upper body (thorax, upper limbs, cephalic extremity). Fontes et al. reported a case of metastatic gastric carcinoma with signet-ring cells (with Krukenberg tumor), an aggressive cancer, whose first clinical sign was UEDVT. The patient was also treated with DOAC for the initial venous thrombusembolism (VTE), which was continued until she died 7 months later, due to rapid deterioration (11).

### 3.2 Anticoagulation therapy in cancer-associated thrombosis

#### 3.2.1 Bleeding risk in cancer-associated thrombosis

Oncologic patients have an increased risk of both venous thromboembolism and bleeding. Therefore, treatment with anticoagulants needs to be precisely indicated to lower the risk for thrombus formation and embolization, but with a minimal elevation in the risk of severe hemorrhagic events (12, 13).

The risk of hemorrhage in cancer-associated thrombosis is multifactorial and results from the cumulative interaction between tumor characteristics, patient-related factors, anticancer therapies, and anticoagulant pharmacology. International guidelines uniformly emphasize that anticoagulant selection should be guided primarily by bleeding risk rather than thrombotic risk alone, recommending DOACs only in carefully selected patients with low hemorrhagic risk, preserved renal and hepatic function, and no clinically relevant drug–drug interactions (14, 15). Meta-analyses of randomized controlled trials have consistently shown that, although DOACs reduce recurrent venous thromboembolism compared with LMWH, they are associated with an increased incidence of clinically relevant non-major bleeding and, in certain subgroups, major bleeding, particularly gastrointestinal hemorrhage (16, 17).

Tumor-related factors are major determinants of bleeding risk. Locally advanced malignancies with vascular invasion or mucosal involvement predispose to hemorrhage through fragile neoangiogenesis, endothelial disruption, and local inflammatory activation. This mechanism explains the higher bleeding rates seen in gastrointestinal or genitourinary luminal cancer patients treated with edoxaban or rivaroxaban in the HOKUSAI-VTE Cancer (18) and SELECT-D studies (19), respectively. In contrast, the CARAVAGGIO study (17). demonstrated that apixaban was not associated with an increased risk of major bleeding compared with dalteparin, supporting the guideline recommendations favoring apixaban in selected patients when considering a DOAC.

Factors associated with the patient further modulate the risk of bleeding and include old age, anemia, renal or hepatic failure, thrombocytopenia, and extremes of body weight. Thrombocytopenia is common in oncology patients and can result from bone marrow infiltration, chemotherapy-induced myelosuppression, or immune-mediated mechanisms. Current International Society on Thrombosis and Haemostasis (ISTH) (20), American Society of Clinical Oncology (ASCO) (21), National Comprehensive Cancer Network (NCCN) (22), European Society of Cardiology (ESC) (23) guidelines recommend full-dose anticoagulation only when platelet counts exceed 50.000/mm³, with LMWH being preferred below this threshold due to more flexible dosing and reversibility.

Anticancer therapies contribute substantially to the risk of bleeding and are a key reason for the cautious use of DOAC. Cytotoxic chemotherapy induces mucosal lesions and epithelial disturbances throughout the gastrointestinal tract, creating a permissive substrate for bleeding under systemic anticoagulation. In addition, many chemotherapeutic and targeted agents interfere with platelet production or function and alter the metabolism of anticoagulants via cytochrome P450 3A4 and P-gp pathways (15, 24). Inhibition of these pathways may increase DOAC plasma concentrations and the risk of bleeding, whereas induction may reduce anticoagulant efficacy and increase thrombotic recurrence. It is important to note that these pharmacokinetic interactions may be accentuated in cancer patients due to sarcopenia, hypoalbuminemia, systemic inflammation, and fluctuations in renal or hepatic function (25).

Taken together, current evidence and guideline recommendations support an individualized approach to anticoagulation in cancer-associated thrombosis, with dynamic reassessment of bleeding risk during cancer treatment. In patients with low bleeding risk and stable clinical parameters, DOACs represent an effective and safe alternative to LMWH, while LMWH remains the preferred treatment in high-risk settings, including active mucosal diseases, severe thrombocytopenia, and complex chemotherapy regimens.

The choice of anticoagulant therapy in our patient was influenced by the unusual localization of thrombosis in the upper limb and by the theoretical bleeding risk associated with tumor infiltration of adjacent vascular structures.

Guidelines suggest that UEDVT may be treated similarly to lower extremity DVT, with DOACs as a reasonable option despite limited direct evidence (20–23).

Although tumoral vascular infiltration was present, the patient was not considered to be at high risk of bleeding, in line with current evidence indicating that tumor-related vascular involvement alone does not necessarily confer an increased hemorrhagic risk in the absence of active mucosal lesions or other major bleeding risk factors.

In our patient, although he has tumor infiltration of vascular structures, he does not have an increased risk of bleeding that would contraindicate the use of DOAC. Platelet counts and coagulation parameters remained within normal limits during observation; renal and hepatic function were preserved; and there was no history of prior bleeding. Furthermore, oncological treatment initially consisted of endocrine therapy, which is not associated with mucosal toxicity or clinically relevant pharmacokinetic interactions with factor Xa inhibitors. Based on this favorable hemorrhagic risk profile and the well-established efficacy of rivaroxaban in reducing recurrent venous thromboembolism in cancer-associated thrombosis, rivaroxaban was selected after initial LMWH therapy without hemorrhagic complications during follow-up.

Nevertheless, anticoagulation was initiated with LMWH, considering the need for invasive diagnostic procedures during hospitalization, as LMWH is preferred in the periprocedural setting due to its shorter half-life and easier reversibility.

#### 3.2.2 Beyond anticoagulation: potential antineoplastic properties of DOACs

Thrombin and factor Xa (significant coagulation factors and the primary targets of DOACs) are involved in tumor initiation and growth, through multiple mechanisms: inflammation, tissue fibrosis, angiogenesis, and metastatic spread. These processes support the potential antineoplastic effect of DOACs (26, 27) Factor Xa inhibitors reduced serum levels of pro-inflammatory cytokines, including tumour necrosis factor (TNF)α, interleukins (IL)-1, IL-6, and IL-8. They negatively impact the expression of monocyte chemoattractant protein 1 (MCP-1) and adhesion molecules, which are involved in leukocyte recruitment and subsequent inflammation (28, 29).

Each factor Xa inhibitor has a specific mechanism underlying its anticancer activity. For example, edoxaban reduces the production of reactive oxygen species (ROS), thereby contributing to its antiangiogenic effect. Tumor proliferation is inhibited by an apixaban-mediated reduction in the proteolytic activity of the factor VII–tissue factor (TF) complex, limiting protease-activating receptor (PAR-2)- 2 activation and the consequent release of TNF microvesicles (TNFα is an essential stimulator of tumor growth). Rivaroxaban distinguishes itself from other DOACs by its antiangiogenic properties and effects on anticancer immunity. It increases the abundance of dendritic cells and cytotoxic T cells at the tumor site, having a synergistic effect with immunotherapy (30, 31).

Although DOACs have shown antineoplastic effects in animal models, their use remains limited to anticoagulant therapy in cancer-associated thrombosis, as evidence in humans is still lacking.

Moreover, the pro-tumorigenic potential is not limited to tissue factor and factor Xa; recent evidence also points to a role of factor XI in modulating tumor biology.

#### 3.2.3 Next-generation anticoagulation: factor XI as a promising therapeutic target

Factor XI (FXI), a 160 kDa serine protease of the intrinsic coagulation pathway, predominantly supports hemostasis by activating factor IX following thrombin-mediated activation. Although FXI's primary hemostatic role involves stabilizing fibrin formation, emerging evidence suggests FXI may directly influence tumor characteristics by modulating migration, cell adhesion, inflammation, immune system evasion, and angiogenesis (32, 33).

Ongoing clinical evaluation includes a phase 2 trial assessing the efficacy and safety of the recombinant humanized monoclonal antibody (mAb) xisomab 3G3 for preventing catheter-related thrombosis in cancer patients undergoing chemotherapy (NCT04465760). Additionally, two phase 2 studies are comparing novel mAbs: the MAGNOLIA trial (NCT05171075) compares abelacimab with dalteparin for VTE prevention in gastrointestinal and genitourinary cancers. In contrast, the ASTER trial (NCT05171049) compares abelacimab to apixaban for CAT (27, 34).

A substantial Phase 3 clinical program is currently underway, comprising two complementary trials—Aster (NCT05171049) and Magnolia (NCT05171075)—enrolling 1,655 and 1,020 patients across over 220 sites in more than 20 countries. These trials represent the most extensive investigations into anticoagulant therapy for CAT. The primary objective is to compare abelacimab with standard care: DOACs in CAT patients and LMWH in GI/GU cancer cohorts. The primary endpoint is the time to the first adjudicated recurrence of venous thromboembolism (RVTE), defined as a new proximal deep vein thrombosis (DVT), new or fatal pulmonary embolism (PE), including sudden unexplained death where PE cannot be excluded. Secondary endpoints include the time to the first major or clinically relevant non-major bleeding (CRNMB) events, and overall net clinical benefit, represented by survival free from RVTE and major or CRNMB events (35).

The Aster trial is a multicenter, randomized, open-label, endpoint-blinded study comparing the effects of intravenous (IV) abelacimab 150 mg administered on day 1 and subcutaneously (SC) monthly thereafter for up to six months, vs. oral apixaban 10 mg twice daily for the first week, then 5 mg twice daily for six months, in patients with confirmed CAT, excluding basal and squamous cell carcinomas of the skin. The Magnolia trial is similarly designed, but focuses on patients with GI and GU cancers for whom DOAC therapy is contraindicated due to high bleeding risk. These patients will receive either abelacimab, as in the Aster trial, or dalteparin, administered SC at 200 IU/kg daily for the first month, followed by 150 IU/kg daily for up to six months. Eligibility requires documented symptomatic or incidental proximal lower extremity DVT or PE, including incidental PE in segmental or larger pulmonary arteries (35).

### 3.3 The length of anticoagulation therapy in cancer-associated thrombosis

The majority of the studies and guidelines recommend anticoagulation therapy in cancer patients for at least 3–6 months after the initial VTE, to prevent recurrence. After 6 months, anticoagulation necessity should be reassessed for each patient, based on malignancy status, risk-benefit ratio, and expert opinion (36).

Studies show that extended anticoagulation beyond 6 months reduces RVTE without significantly increasing major bleeding, supporting continuation in patients with active malignancy (37–39).

The American Society of Hematology (ASH 2021) and American Society of Clinical Oncology (ASCO 2023) guidelines both endorse long-term or indefinite anticoagulation while the cancer remains active, preferably with DOACs or LMWHs, depending on renal/hepatic function and bleeding risk (21, 22, 40, 41). ESC guidance also acknowledges the potential role of dose-adjusted or reduced-dose anticoagulation during extended treatment, particularly in selected patients in whom bleeding risk outweighs thrombotic risk, although robust prospective data remain limited (23).

Bakht et al. (42) addressed the question of reducing DOAC doses in long-term anticoagulation, finding that reduced DOAC doses do not differ significantly from conventional doses in lowering the risk of RVTE in patients with active cancer. A reduced-dose approach offered similar protection against RVTE, with a surprisingly relevant reduction in incidental/asymptomatic VTE events (OR = 0.31, 95% CI 0.14–0.69, *p* = 0.004). In terms of hemorrhagic risk, there was no significant reduction for either major bleeding or CRNMB between reduced-dose and full-dose regimens; however, composite analysis of bleeding events (considering both major and non-major events) demonstrated a statistically significant reduction in risk with half-doses of anticoagulants (OR = 0.69, 95% CI 0.55–0.88, *p* = 0.002). These results offer an alternative, efficient, but possibly safer, approach to long-term anticoagulation regimens (with reduced doses) in cancer patients, especially those with a greater risk of hemorrhagic events. Nevertheless, further studies are needed to confirm this information (42).

### 3.4 Interventional therapy in UEDVT

Mechanical reperfusion is currently recommended for axillar-subclavian UEDVT in severely symptomatic patients, with relevant thrombus burden, recent thrombosis (< 14 days), good functional status and life expectancy, and low hemorrhagic risk. Even after catheter-directed thrombolysis, anticoagulation should be continued post-procedure (9).

An expert opinion is strongly recommended before thrombolytic therapy in UEDVT, and the procedure should be done in experienced health-care centers. It requires careful patient selection, screening for brain metastasis (especially in high-risk patients), and possible adjunctive therapy (e.g., mechanical thrombectomy, to reduce the dose of thrombolytic agent) (12, 13).

## 4 Conclusions

UEDVT is frequently associated with malignancy, representing occasionally the first clinical manifestation of an underlying tumor DOACs are increasingly replacing LMWH as the preferred treatment for cancer-associated DVT. The duration of anticoagulation should be extended beyond six months if the malignancy remains active, while dose reduction after this period is recommended only in carefully selected cases.

Emerging data suggest that monoclonal antibodies targeting FXI(a) hold promise in the management of cancer-associated DVT and may also influence tumor biology by modulating cell adhesion, migration, inflammation, immune evasion, and angiogenesis.

## Data Availability

The raw data supporting the conclusions of this article will be made available by the authors, without undue reservation.
